# Supplementary material for: On the contribution of work or heat in exchanged energy via interaction in open bipartite quantum systems
Source: Sci Rep. 2023 Jan 4;13:160. doi: 10.1038/s41598-022-27156-0 (PMC9813259; doi:10.1038/s41598-022-27156-0)
Supplement: Supplementary file 1 — Supplementary Information. [file 41598_2022_27156_MOESM1_ESM.pdf]

On the Contribution of Work or Heat in Exchanged Energy via Interaction in Open bipartite Quantum Systems

B. Ahmadi[1,*], S. Salimi[2], and A. S. Khorashad[2]

[1]International Centre for Theory of Quantum Technologies, University of Gdansk, Jana Bażyńskiego 1A, 80-309 Gdansk, Poland

[2]Department of Physics, University of Kurdistan, P.O.Box 66177-15175, Sanandaj, Iran
[*]borhan.ahmadi@ug.edu.pl

Supplementary Note 1

The infinitesimal change in the eigenvectors of the state of the system $\rho_A(t)$ in Eq. (13) is achieved as

$$\begin{aligned} d(|\psi_i(t)\rangle\langle\psi_i(t)|) &= |\psi_i(t+dt)\rangle\langle\psi_i(t+dt)| - |\psi_i(t)\rangle\langle\psi_i(t)| \\ &= U(dt)|\psi_i(t)\rangle\langle\psi_i(t)|U^\dagger(dt) - |\psi_i(t)\rangle\langle\psi_i(t)|, \end{aligned} \tag{1}$$

where $U(dt)$ is the unitary operator transforming the orthogonal basis $\{|\psi_i(t)\rangle\}_{i=1}^d$ to another orthogonal basis $\{|\psi_i(t+dt)\rangle\}_{i=1}^d$ [S2]. Using Eq. (1) and Eq. (6) of the main text we have

$$\sum_{i=1}^{d} p_i(t) d(|\psi_i(t)\rangle\langle\psi_i(t)|) = U(dt)\rho_A(t)U^\dagger(dt) - \rho_A(t). \tag{2}$$

Eq. (2) is the unitary part of the total change in the state of the system $\rho_A(t)$. Now substituting Eq. (2) into Eq. (13) of the main text the second term on the right hand side of Eq. (13) reads

$$tr\{\sum_{i=1}^{d} p_i(t) d(|\psi_i(t)\rangle\langle\psi_i(t)|) H_A(t)\} = tr\{d\rho_A^U(t) H_A(t)\}, \tag{3}$$

in which $d\rho_A^U(t) \equiv U(dt)\rho_A(t)U^\dagger(dt) - \rho_A(t)$. Thus Eq. (3) is in fact the energy which is unitarily exchanged between the two quantum systems through the interaction.

Supplementary Note 2

The average of the internal energy of system A at time t is defined as[S1]

$$\begin{aligned} \langle E_A(t)\rangle &= tr\{\rho_A(t) H_A(t)\} \\ &= tr\{\sum_{i=1}^{d} p_i(t)|\psi_i(t)\rangle\langle\psi_i(t)| H_A(t)\} \\ &= \sum_{i=1}^{d} p_i(t)\langle\psi_i(t)|H_A(t)|\psi_i(t)\rangle. \end{aligned} \tag{4}$$

On the other hand

$$H_A(t) = \sum_j E_j(t)|E_j(t)\rangle\langle E_j(t)|, \tag{5}$$

therefore

$$\begin{aligned}\langle E_A(t)\rangle &= \sum_{i=1}^{d}\sum_{j} p_i(t)\,E_j(t)|\langle\psi_i(t)|E_j(t)\rangle|^2\\ &= \sum_{j} q_j(t)\,E_j(t), \end{aligned} \tag{6}$$

where

$$\begin{aligned}q_j(t) &= \sum_{i=1}^{d} p_i(t)\,|\langle\psi_i(t)|E_j(t)\rangle|^2,\\ &= \sum_{i=1}^{d} p_i(t)\,R_{i\to j}(t), \end{aligned} \tag{7}$$

with $R_{i\to j}(t)$ the transition probability from the eigenbasis $|\psi_i(t)\rangle$ to the eigenbasis $|E_j(t)\rangle$. The connection between $p_i(t)$ and $q_i(t)$ is given through Eq. (7). Now from Eqs. (6) and (7) we have

$$\langle E_A(t)\rangle = \sum_{i=1}^{d}\sum_{j} p_i(t)\,R_{i\to j}(t)\,E_j(t). \tag{8}$$

Then

$$d\langle E_A(t)\rangle = \underbrace{\sum_{i=1}^{d}\sum_{j} dp_i(t)\,R_{i\to j}(t)\,E_j(t)}_{đ\langle Q_A(t)\rangle} + \underbrace{\sum_{i=1}^{d}\sum_{j} p_i(t)\,d(R_{i\to j}(t)\,E_j(t))}_{đ\langle R(t)\rangle}.$$

Hence we have

$$đ\langle Q_A(t)\rangle = \sum_{i=1}^{d}\sum_{j} dp_i(t)\,R_{i\to j}(t)\,E_j(t), \tag{9}$$

$$đ\langle W_A(t)\rangle = \sum_{i=1}^{d}\sum_{j} p_i(t)\,dR_{i\to j}(t)\,E_j(t) + \sum_{i=1}^{d}\sum_{j} p_i(t)\,R_{i\to j}(t)\,dE_j(t),$$

which means that two terms contribute to $dW(t)$, one is originating from the variation of transition probabilities (the first term) and the other originates from the variations of the energy levels (the second term).

References

S1. Gemmer, J., Michel, M. & Mahler, G. Quantum thermodynamics: Emergence of thermodynamic behavior within composite quantum systems, vol. 784 (Springer, 2009).

S2. Heinosaari, T. & Ziman, M. The mathematical language of quantum theory: from uncertainty to entanglement (Cambridge University Press, 2011).
